# The Performance of GPT-3.5, GPT-4, and Bard on the Japanese National Dentist Examination: A Comparison Study

**DOI:** 10.7759/cureus.50369

**Published:** 2023-12-12

**Authors:** Keiichi Ohta, Satomi Ohta

**Affiliations:** 1 School of Medicine, Kobe University, Kobe, JPN; 2 Dentistry, Dentist of Mama and Kodomo, Kobe, JPN

**Keywords:** japan, national dentist examination, artificial intellinge in dentistry, google bard, chatgpt-3.5, chatgpt-4

## Abstract

Purpose

This study aims to evaluate the performance of three large language models (LLMs), the Generative Pre-trained Transformer (GPT)-3.5, GPT-4, and Google Bard, on the 2023 Japanese National Dentist Examination (JNDE) and assess their potential clinical applications in Japan.

Methods

A total of 185 questions from the 2023 JNDE were used. These questions were categorized by question type and category. McNemar's test compared the correct response rates between two LLMs, while Fisher’s exact test evaluated the performance of LLMs in each question category.

Results

The overall correct response rates were 73.5% for GPT-4, 66.5% for Bard, and 51.9% for GPT-3.5. GPT-4 showed a significantly higher correct response rate than Bard and GPT-3.5. In the category of essential questions, Bard achieved a correct response rate of 80.5%, surpassing the passing criterion of 80%. In contrast, both GPT-4 and GPT-3.5 fell short of this benchmark, with GPT-4 attaining 77.6% and GPT-3.5 only 52.5%. The scores of GPT-4 and Bard were significantly higher than that of GPT-3.5 (p<0.01). For general questions, the correct response rates were 71.2% for GPT-4, 58.5% for Bard, and 52.5% for GPT-3.5. GPT-4 outperformed GPT-3.5 and Bard (p<0.01). The correct response rates for professional dental questions were 51.6% for GPT-4, 45.3% for Bard, and 35.9% for GPT-3.5. The differences among the models were not statistically significant. All LLMs demonstrated significantly lower accuracy for dentistry questions compared to other types of questions (p<0.01).

Conclusions

GPT-4 achieved the highest overall score in the JNDE, followed by Bard and GPT-3.5. However, only Bard surpassed the passing score for essential questions. To further understand the application of LLMs in clinical dentistry worldwide, more research on their performance in dental examinations across different languages is required.

## Introduction

The advancement of artificial intelligence, particularly in the realm of large language models (LLMs), has been remarkable in recent years. These models, capable of generating human-like sentences by processing extensive text data, are increasingly utilized in various domains for understanding context, answering questions, and facilitating language translation [1]. In dentistry, LLMs have potential applications in dental telemedicine, clinical decision-making, administrative work, patient education, and dental school education [2].

The integration of LLMs into clinical practice has garnered significant interest in the medical field. Researchers have evaluated their proficiency by testing them with national and board medical examinations on multiple-choice questions (MCQs) [3]. One of the most extensively researched LLMs is ChatGPT, the Chat Generative Pre-Trained Transformer (OpenAI, San Francisco, California), released in November 2022. It includes advanced versions like GPT-3.5 and GPT-4 [4]. In March 2023, Google released Google Bard (Google LLC, Mountain View, California), characterized by its internet search function, providing access to current data [5]. In English-speaking countries, GPT-4 has been reported to meet the passing criteria for both the United States Medical Licensing Examination and the United Kingdom Medical Licensing Assessment [6-8]. Comparative studies between GPT and Bard have demonstrated GPT-4's superiority in answering several professional questions [9-11].

In Japan, GPT-4 showed proficiency in passing the Japanese Medical Licensing Examination (JMLE), the Japanese National Examination for Pharmacists (JNEP), the Japanese National Nursing Examination (JNNE), and the official board examination of the Japan Radiology Society [12-15]. However, the performance of LLMs on the Japanese National Dentist Examination (JNDE) remains unexplored, and research in the field of dentistry is limited. One study reported that GPT-3.5 did not pass the Iranian Endodontics Specialist Board [16].

This study aims to evaluate the performance of three LLMs on the JNDE and assess their potential clinical applications in Japan.

## Materials and methods

Large language models

This study evaluated three LLMs: GPT-3.5, GPT-4 (version: August 3, 2023), and Bard. Two models of GPT were assessed: GPT-3.5, which is freely available, and GPT-4, a high-performance model accessible via a monthly subscription.

Japanese National Dentist Examination 

The 2023 JNDE (116th) was used for testing and analysis [17]. It includes 80 essential questions, Domain A with 100 general questions, and Domain B with 160 questions, divided equally between special questions and practical clinical questions. Among these, three questions required calculations. Essential questions assess the fundamental knowledge and skills necessary for a dentist, general questions cover basic medicine, epidemiology, and general dentistry, while special questions focus on specialized dentistry areas. Practical clinical questions pertain to examinations, diagnoses, treatments, and procedural sequences in clinical dentistry cases. These questions often include figures or tables, contrasting with the text-only nature of other question types. Scoring involves 3 points per practical clinical question and 1 point per other question. The minimum passing scores for the 2023 JNDE are 80% for essential questions, 65.6% for Domain A, and 68.9% for Domain B [17]. Due to non-disclosure by the Ministry of Health, Labor and Welfare (MHLW) regarding the classification of questions into Domain A or B, assessment of scores for these domains was not feasible.

Exclusion criteria

The exclusion criteria encompassed 15 questions deemed ineligible for scoring by the MHLW, 185 questions containing images or tables, and 2 questions requesting the latest Japanese dental statistics. Consequently, the final analysis included 185 questions.

Classification of questions

To evaluate the LLMs' capability to answer professional dental questions in Japanese, questions requiring specialized dentistry knowledge were categorized as dentistry questions. This classification was independently conducted by two experienced dentists (K.O. and S.O.) who did not know the correct answer, with only consensus questions being included. Questions answerable without specific dentistry knowledge, such as those related to anesthesiology, internal medicine, and otorhinolaryngology, were excluded.

Prompt engineering

The original JNDE questions, presented in Japanese, were manually inputted into the LLMs' chat interfaces, one at a time, to obtain responses. Prior to each question, the following instruction in Japanese was entered: “You are a candidate for the Japanese National Dentist Examination. Please answer the following question.” A response was deemed "correct" if it matched the official answers provided by the MHLW [18]. This analysis was conducted from September 1 to 3, 2023.

Data analysis

For statistical analysis, standard descriptive statistics were utilized. In accordance with previous studies, McNemar's test compared the correct response rates between two LLMs, while Fisher’s exact test evaluated the performance of LLMs in each question category [12,13]. These tests were two-tailed, with a p-value of less than 0.05 indicating statistical significance. All statistical analyses were performed using R version 3.5.1 (R Foundation for Statistical Computing, Vienna, Austria).

Ethical considerations

This study exclusively used publicly available internet data and did not involve human subjects. Consequently, it was exempt from specific ethical considerations.

## Results

**Figure 1:**
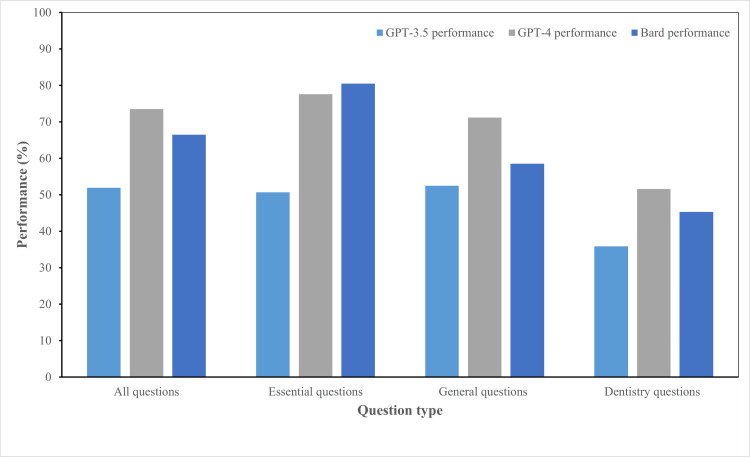
The performance of GPT-3.5, GPT-4, and Bard on each question type

In the 2023 JNDE, which initially comprised 360 questions, only 185 questions were considered for this study after excluding those with images or diagrams. Among these, 67 were identified as essential questions and 64 as dentistry questions. The response accuracy of the three LLMs, i.e., GPT-4, GPT-3.5, and Bard, was evaluated across these questions (Figure 1).

The overall correct response rates were 73.5% (136/185) for GPT-4, 66.5% (123/185) for Bard, and 51.9% (96/185) for GPT-3.5 (Table 1). GPT-4 showed a significantly higher correct response rate than Bard and GPT-3.5.

**Table 1:** The performance of GPT-3.5, GPT-4, and Bard on each question type GPT: Generative Pre-trained Transformer

Question type	Number of questions	Correct response rates (%)			p value		
		GPT-3.5	GPT-4	Bard	GPT-3.5 vs. GPT-4	GPT-3.5 vs. Bard	GPT-4 vs. Bard
All questions	185	96(51.9)	136(73.5)	123(66.5)	<0.001	0.0037	0.039
Essential questions	67	34(50.7)	52(77.6)	54(80.5)	0.001	0.003	0.803
General questions	118	62(52.5)	84(71.2)	69(58.5)	<0.001	0.461	0.004
Dentistry questions	64	23(35.9)	33(51.6)	29(45.3)	0.0953	0.3447	0.6767

In the category of essential questions, Bard achieved a correct response rate of 80.5% (64/67), surpassing the passing criterion of 80%. In contrast, both GPT-4 and GPT-3.5 fell short of this benchmark, with GPT-4 attaining 77.6% (52/67) and GPT-3.5 only 52.5% (34/67). Notably, the scores of GPT-4 and Bard were significantly higher than the score of GPT-3.5 (p<0.01).

For general questions, the correct response rates were 71.2% (84/118) for GPT-4, 58.5% (69/118) for Bard, and 52.5% (62/118) for GPT-3.5. Here again, GPT-4 outperformed GPT-3.5 and Bard (p<0.01).

When examining dentistry questions specifically, the correct response rates were 51.6% (33/64) for GPT-4, 45.3% (29/64) for Bard, and 35.9% (23/64) for GPT-3.5. However, in this category, the differences among the models were not statistically significant.

A comparative analysis (Table 2) of the percentage of correct answers for dentistry versus other questions revealed a noteworthy trend. All LLMs demonstrated significantly lower accuracy for dentistry questions compared to other types of questions (p<0.01).

**Table 2:** The percentage of correct answers for dentistry versus other questions LLM: Large Language Model, GPT: Generative Pre-trained Transformer

LLM	Correct response rates of dentistry questions vs. others (%)	p value
GPT-3.5	35.9 vs. 57.9	0.005
GPT-4	51.6 vs. 85.1	<0.001
Bard	45.3 vs 76.0	<0.001

## Discussion

In this study, we evaluated the correct response rates of GPT-3.5, GPT-4, and Bard on the 2023 JNDE. GPT-4 achieved the highest overall score, followed by Bard and GPT-3.5. This aligns with previous findings where GPT-4 outperformed GPT-3.5 and Bard in terms of overall correct response rates [9,11,15]. Because detailed scoring criteria were not announced for all but the essential questions, we were unable to assess whether the LLMs met the JNDE's passing criteria.

In essential questions, Bard barely achieved the passing criteria, whereas GPT-3.5 and GPT-4 did not satisfy the required scores. Previous studies assessing GPT-4 and GPT-3.5 on the JMLE and JNNE found that GPT-4 met the passing criteria, unlike GPT-3.5 [12,13]. However, in our study, even GPT-4 did not surpass the passing score for the JNDE's essential questions, albeit closely. This could be attributed to the increasing difficulty of the JNDE. In 2006, The MHLW considered the current number of dentists in Japan to be excessive in relation to demand [19]. To control the increase in the number of new dentists, MHLW has taken measures to raise the passing criteria for the JNDE. In fact, the pass rates for the JNDE for the last 10 years have all been in the 60% range. In contrast, the pass rates for the JMLE and JNNE have been almost 90% [20-22]. Additionally, LLMs scored low percentages of correct answers for dentistry questions. Compared to the JMLE and JNNE, the low percentage of LLM scores on the essential questions for the JNDE was probably due to the low percentage of correct answers in the dentistry question. One potential cause of low performance for the dentistry questions may be due to the fact that the amount of medical literature in Japanese, especially dentistry, is smaller than that in English, resulting in a smaller amount of data to learn in Japanese [15,23]. To assess the applicability of LLMs in clinical dentistry globally, more studies on their performance in national dental examinations across various languages are needed.

Research on the performance of LLMs in dentistry questions is limited. Two studies on the use of GPTs for endodontic questions in English reported that the models did not meet specific criteria [16,24]. Suárez's study indicated that despite high consistency (85.4%), the correct answer percentage for GPT-4 in endodontic questions was only 57.3%, suggesting that these models cannot currently replace clinical decision-making by dentists [24]. Moreover, the ability to interpret visual findings and radiographic images is essential to the practice of dentistry. The JNDE assesses these skills in the practical clinical questions, which account for approximately 50% of the total score, indicating that visual information is highly valued. The implementation of the image recognition function of LLMs is awaited to study the clinical application of LLMs in dentistry.

Healthcare providers considering the use of LLMs in clinical practice should be aware of "hallucinations," a phenomenon where LLMs present incorrect or fictional information as correct. GPT-4 has been reported to be less prone to "hallucinations" in neurosurgical MCQs, suggesting potential advancements in AI to further reduce these occurrences in the medical field [9].

This study has several limitations. Firstly, the LLMs were tested only once, despite their potential for varying responses. Multiple trials could provide a more accurate assessment of response consistency. Several studies have reported varying consistency levels for GPT responses, ranging from 36.4 to 88.8% [10,23,25]. To accurately assess the performance of LLMs, we need to conduct two or more trials and evaluate the consistency. Secondly, the LLMs were not tested on imaging questions, which are crucial for practical clinical applications. As image analysis capabilities improve, a new assessment incorporating these questions will be necessary. Thirdly, the rapid advancement in LLM technology means that the responses to the JNDE questions may change, so each test was conducted within a single day to mitigate this issue. Fourth, the classification of dentistry questions potentially introduced selection bias. Therefore, caution should be exercised in interpreting the finding that LLMs had lower percentages of correct responses to dentistry questions. Fifth, the quality of GPT responses varies depending on the prompt, making it difficult to simply compare GPTs and Bard scores [26]. Despite these limitations, we believe that this study could be a valuable evaluation of the potential use of LLM in dental medicine in Japan.

## Conclusions

In conclusion, our study demonstrated that GPT-4 achieved the highest overall score in the JNDE, followed by Bard and GPT-3.5. However, only Bard surpassed the passing score for essential questions. To further understand the application of LLMs in clinical dentistry worldwide, more research on their performance in dental examinations across different languages is required.

## References

[1] Clusmann J, Kolbinger FR, Muti HS (2023). The future landscape of large language models in medicine. Commun Med (Lond).

[2] Eggmann F, Weiger R, Zitzmann NU, Blatz MB (2023). Implications of large language models such as ChatGPT for dental medicine. J Esthet Restor Dent.

[3] Newton PM, Xiromeriti M (2023). ChatGPT performance on MCQ exams in higher education: a pragmatic scoping review. EdArXiv.

[4] (2023). ChatGPT. https://openai.com/blog/chatgpt/.

[5] (2023). Bard. https://bard.google.com.

[6] Kung TH, Cheatham M, Medenilla A (2023). Performance of ChatGPT on USMLE: potential for AI-assisted medical education using large language models. PLOS Digit Health.

[7] Gilson A, Safranek CW, Huang T, Socrates V, Chi L, Taylor RA, Chartash D (2023). How does ChatGPT perform on the United States Medical Licensing Examination? The implications of large language models for medical education and knowledge assessment. JMIR Med Educ.

[8] Lai UH, Wu KS, Hsu TY, Kan JK (2023). Evaluating the performance of ChatGPT-4 on the United Kingdom Medical Licensing Assessment. Front Med (Lausanne).

[9] Ali R, Tang OY, Connolly ID (2023). Performance of ChatGPT, GPT-4, and Google Bard on a neurosurgery oral boards preparation Question Bank. Neurosurgery.

[10] Koga S (2023). Exploring the pitfalls of large language models: inconsistency and inaccuracy in answering pathology board examination-style questions. Pathol Int.

[11] Iannantuono GM, Bracken-Clarke D, Karzai F, Choo-Wosoba H, Gulley JL, Floudas CS (2023). Comparison of large language models in answering immuno-oncology questions: a cross-sectional study. Oncology.

[12] Takagi S, Watari T, Erabi A, Sakaguchi K (2023). Performance of GPT-3.5 and GPT-4 on the Japanese Medical Licensing Examination: comparison study. JMIR Med Educ.

[13] Kaneda Y, Takahashi R, Kaneda U (2023). Assessing the performance of GPT-3.5 and GPT-4 on the 2023 Japanese Nursing Examination. Cureus.

[14] Kunitsu Y (2023). The potential of GPT-4 as a support tool for pharmacists: analytical study using the Japanese National Examination for Pharmacists. JMIR Med Educ.

[15] Toyama Y, Harigai A, Abe M, Nagano M, Kawabata M, Seki Y, Takase K (2023). Performance evaluation of ChatGPT, GPT-4, and Bard on the official board examination of the Japan Radiology Society. Jpn J Radiol.

[16] Farajollahi M, Modaberi A (2023). Can ChatGPT pass the “Iranian Endodontics Specialist Board” exam?. Iran Endod J.

[17] (2023). The 116th National Dentist Examination (Article in Japanese). https://www.mhlw.go.jp/seisakunitsuite/bunya/kenkou_iryou/iryou/topics/tp230524-02.html.

[18] (2023). Announcement of successful candidates for the 116th National Dentist Examination (Article in Japanese). https://www.mhlw.go.jp/general/sikaku/successlist/2023/siken02/about.html.

[19] (2023). Recent trends in dental health care (Article in Japanese). https://www.mhlw.go.jp/content/10804000/000742124.pdf.

[20] (2023). Announcement of past successful candidates for the National Dentist Examination (Article in Japanese). http://Https://Www.Mhlw.Go.Jp/Stf/Shingi/Shingi-Idou_127793.Html.

[21] (2023). Announcement of past successful candidates for the National Nursing Examination (Article in Japanese). http://Https://Www.Mhlw.Go.Jp/Stf/Shingi/Shingi-Idou_127797.Html.

[22] (2023). Announcement of past successful candidates for the National Medical Licensing Examination (Article in Japanese). http://Https://Www.Mhlw.Go.Jp/Stf/Shingi/Shingi-Idou_127788.Html.

[23] Haze T, Kawano R, Takase H, Suzuki S, Hirawa N, Tamura K (2023). Influence on the accuracy in ChatGPT: differences in the amount of information per medical field. Int J Med Inform.

[24] Suárez A, Díaz-Flores García V, Algar J, Gómez Sánchez M, Llorente de Pedro M, Freire Y (2023). Unveiling the ChatGPT phenomenon: evaluating the consistency and accuracy of endodontic question answers. Int Endod J.

[25] Beaulieu-Jones BR, Shah S, Berrigan MT, Marwaha JS, Lai SL, Brat GA (2023). Evaluating capabilities of large language models: performance of GPT4 on surgical knowledge assessments. medRxiv.

[26] Chen Y, Zhao C, Yu Z, McKeown K, He H (2023). On the relation between sensitivity and accuracy in in-context learning. arXiv.

